# Automatic Brain Tissue and Lesion Segmentation and Multi-Parametric Mapping of Contrast-Enhancing Gliomas without the Injection of Contrast Agents: A Preliminary Study

**DOI:** 10.3390/cancers16081524

**Published:** 2024-04-17

**Authors:** Jing Liu, Angela Jakary, Javier E. Villanueva-Meyer, Nicholas A. Butowski, David Saloner, Jennifer L. Clarke, Jennie W. Taylor, Nancy Ann Oberheim Bush, Susan M. Chang, Duan Xu, Janine M. Lupo

**Affiliations:** 1 Department of Radiology and Biomedical Imaging, University of California San Francisco, San Francisco, CA 94143, USA; angela.jakary@ucsf.edu (A.J.); duan.xu@ucsf.edu (D.X.); 2 Department of Neurological Surgery, University of California San Francisco, San Francisco, CA 94143, USA; nicholas.butowski@ucsf.edu (N.A.B.); jennifer.clarke@ucsf.edu (J.L.C.); susan.chang@ucsf.edu (S.M.C.); 3 Radiology Service, VA Medical Center, San Francisco, CA 94121, USA; 4 Department of Neurology, University of California San Francisco, San Francisco, CA 94143, USA; 5 UCSF/UC Berkeley Graduate Program in Bioengineering, University of California San Francisco and Berkeley, San Francisco, CA 94143, USA

**Keywords:** gliomas, multi-contrast, automatic lesion detection, two-compartment model, multi-parametric mapping, macromolecular proton fraction

## Abstract

**Simple Summary:**

Standard clinical brain tumor magnetic resonance imaging (MRI) exams require contrast injection and multiple structural MRI sequences, acquired individually in 30 min. This study developed a single 6 min sequence and automatic processing strategy for multi-contrast whole-brain imaging to achieve the lesion detection of gliomas without the use of a contrast agent, which has the potential to significantly improve clinical imaging workflows and provide substantial benefits to patients for which gadolinium is contraindicated. Automatically segmented tumor lesions from fourteen patients with contrast-enhancing gliomas were comparable to manually defined lesions from conventional T2-FLAIR (Fluid-Attenuated Inversion Recovery) and T1-post-contrast imaging with contrast administration. The T2-hyperintensity lesion could be further separated into two components, likely demarcating a more infiltrative tumor region within the edema. Multi-parametric mapping based on multi-compartment modeling allowed for quantitative lesion characterization.

**Abstract:**

This study aimed to develop a rapid, 1 mm^3^ isotropic resolution, whole-brain MRI technique for automatic lesion segmentation and multi-parametric mapping without using contrast by continuously applying balanced steady-state free precession with inversion pulses throughout incomplete inversion recovery in a single 6 min scan. Modified k-means clustering was performed for automatic brain tissue and lesion segmentation using distinct signal evolutions that contained mixed T1/T2/magnetization transfer properties. Multi-compartment modeling was used to derive quantitative multi-parametric maps for tissue characterization. Fourteen patients with contrast-enhancing gliomas were scanned with this sequence prior to the injection of a contrast agent, and their segmented lesions were compared to conventionally defined manual segmentations of T2-hyperintense and contrast-enhancing lesions. Simultaneous T1, T2, and macromolecular proton fraction maps were generated and compared to conventional 2D T1 and T2 mapping and myelination water fraction mapping acquired with MAGiC. The lesion volumes defined with the new method were comparable to the manual segmentations (r = 0.70, *p* < 0.01; *t*-test *p* > 0.05). The T1, T2, and macromolecular proton fraction mapping values of the whole brain were comparable to the reference values and could distinguish different brain tissues and lesion types (*p* < 0.05), including infiltrating tumor regions within the T2-lesion. Highly efficient, whole-brain, multi-contrast imaging facilitated automatic lesion segmentation and quantitative multi-parametric mapping without contrast, highlighting its potential value in the clinic when gadolinium is contraindicated.

## 1. Introduction

Gliomas, the most common intra-axial primary brain tumors in adults with 24,530 new cases expected to be diagnosed in the United States in 2023, are heterogeneous and highly infiltrative, with poorly defined margins [1,2,3]. The median survival time ranges from 7 to 10 years and, for patients with lower-grade, isocitrate dehydrogenase (IDH)-mutant tumors, to 15–18 months for more aggressive IDH-wildtype glioblastomas. Since gliomas are highly infiltrative with poorly defined margins, surgical resection of all tumor cells is nearly impossible, with local recurrence being the most common mode of tumor progression [4]. Structural magnetic resonance imaging (MRI), including pre- and post-contrast T1-weighted, T2-weighted, and T2-FLAIR (Fluid-Attenuated Inversion Recovery) imaging, is critical for surgical and radiation planning, monitoring response to therapy, and determining progression [5]. Although 30–50% of patients undergo gross total resection of the contrast-enhancing lesion (CEL) depicted on post-contrast T1-weighted images, the infiltrative tumor persists in the surrounding T2-hyperintense non-enhancing lesion (T2L) and represents a diagnostic challenge to clinician and automatic segmentation tools. Although T2L is thought to encompass the majority of tumor, it also reflects areas of edema and gliosis. This situation is even more complex after treatment due to changes in the blood–brain barrier that increase the extent of edema and lead to the formation of gliosis, all of which may be confused with tumor progression. This is a major problem in defining treatment effects and rendering traditional markers of tumor burden like the CEL unreliable markers of response, especially in the case of anti-angiogenic therapies, which normalize the vasculature and repair blood–brain barrier breakdown [6].

Despite these limitations with respect to the definition of tumor burden based on structural imaging alone, the Response Assessment in Neuro-Oncology (RANO) criteria for patients with glioma enrolled in clinical trials still depend upon early changes in tumor size based on structural MRI to evaluate the response [7,8]. According to the most recent set of criteria introduced in 2023 [9], progression is defined as a 25% increase in the cross-sectional diameter of the CEL on post-contrast T1-weighted MR images for contrast-enhancing tumors and of the T2L for lower-grade non-enhancing gliomas. Although the limitations of using the cross-sectional diameter as a metric for response assessment are widely acknowledged and there is an increasing trend towards volumetrics, there remains challenges with currently available software, including variability in the results and added costs, complexity, and logistical challenges. Exacerbating this problem is that nearly all automated techniques rely on imaging information from four structural MRI sequences (T1-weighted pre/post-gad, T2-weighted/T2-FLAIR sequences) and deep learning methodologies for inference [10,11], which are mostly applied in the newly diagnosed, pre-operative setting [10,11,12,13,14], with little consistency in the tumor sub-compartments under consideration. Only a few studies to date have attempted to investigate the potential of a fully automatic segmentation method to longitudinally track changes in tumor volume [15,16,17]. There is a need to obtain more objective and robust measurements of tumor burden that are automatically generated by the sequence and more easily compared over time for better understanding therapeutic efficacy and impacting timely treatment decisions.

Quantitative tools that probe the underlying cellular properties, chemical composition, and biophysical mechanisms of the disease to more accurately delineate tumor margins from the areas of infiltrative tumor within the edema based on a T2 signal have been shown to be beneficial for evaluating longitudinal changes in tumor burden, early response to therapy, and prognosis [18,19,20,21,22,23,24,25,26]. In these studies, high-grade lesions with larger percentages of longer T2 values obtained from quantitative parametric mapping signifying more edema and less infiltrative tumor cells improved the outcomes in terms of both progression-free and overall survival. Although tissue characterization through the parametric mapping of relaxation parameters is used in a broad range of applications, T1 and T2 maps are typically estimated separately, ignoring the tissue relaxation effect from the other component, resulting in inaccurate quantification. Simultaneous, multi-parametric mapping (such as T1/T2/proton density) with balanced steady-state free precession (bSSFP) acquisition during inversion recovery (IR) [27] and MR Fingerprinting (MRF) methods that acquire non-steady-state signals (2D imaging) after an inversion pulse [10] provide comprehensive mapping with inherently improved accuracy. The IR-bSSFP technique uses closed-form equations for deriving tissue parameters while MRF derives tissue parameters by matching the acquired signal to simulated signal evolution based on Bloch equations. However, since both methods require a full recovery of magnetization after each inversion pulse and acquisition train, 3D acquisitions in the brain typically have relatively long T1 relaxation times, which is time consuming. MRF mitigates this drawback by highly accelerating data acquisition. By continuously acquiring data with bSSFP readouts during incomplete IR, combined with an efficient undersampling scheme and advanced reconstruction, significant improvements in scan efficiency are achievable. Although bSSFP can suffer from considerable signal loss in certain tissues due to magnetization transfer (MT) effects, which undergo dipolar interactions and chemical exchange both with macromolecular protons and those in the free water pool, these unwanted effects can provide an additional contrast mechanism that is valuable for assessing the underlying tissue composition, reflecting macromolecular protons which are usually “invisible” with conventional MRI sequences.

This study aimed to address these barriers within the conventional structural MRI of gliomas by developing a novel quantitative single-scan sequence with an automatic segmentation strategy that exploits voxel-wise signal evolution, resulting in multiple contrast images, quantitative parametric maps (T1-, T2-, and macromolecular fraction) with potentially greater sensitivities to the infiltrating tumor, and segmented masks of lesion and brain tissue compartments (white matter, grey matter, cerebrospinal fluid, contrast-enhancing lesion, non-enhancing T2-lesion components, and necrosis) without the need for contrast agent injection. The performance of this methodology was assessed in treated patients with enhancing gliomas in terms of its ability to achieve the following: (i) segment lesions similar to manually drawn regions from clinically acquired sequences; (ii) produce parametric maps with comparable values to the conventional measurements; and (iii) further separate regions of shorter T2-components which are likely reflective of more infiltrating tumor from the edema within the non-enhancing lesion.

## 2. Materials and Methods

In this section, we will first introduce a new MRI sequence design, an approach for the optimization of scan parameters, and a description of the advanced sampling and reconstruction strategy for this specific sequence design. The second part of this section then describes image processing for brain tissue and lesion segmentation, deriving multi-parametric mapping, and the validation of the technique in patients with contrast-enhancing glioblastomas.

### 2.1. IIR-bSSFP Acquisition

**Figure 1:**
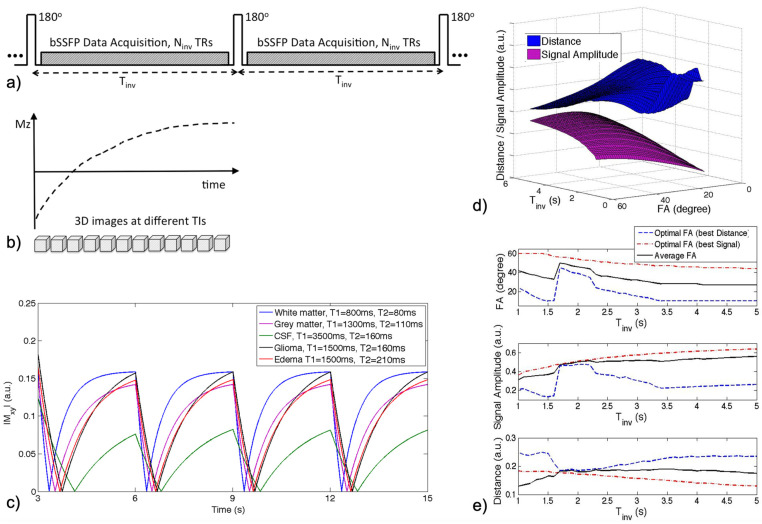
(**a**) IIR-bSSFP acquisition. A nonselective inversion pulse is applied followed by a fixed number of segmented bSSFP acquisitions (IR interval—T_inv_). (**b**) With continuous data acquisition, a series of 3D images is reconstructed at different TIs. (**c**) Simulated signal evolutions of different brain tissues with assumed T1/T2 values and scan parameters (T_inv_ = 3 s, flip angle FA = 30°, TR = 4 ms). (**d**) Averaged signal amplitudes and Euclidean distances of all brain tissues, simulated with a series of FA and T_inv_. (**e**) Given T_inv_, the optimal FA is identified to maximize the signal amplitude to optimize the signal-to-noise level (red dashed curves) and maximize the distance to optimize tissue contrast (blue dashed curves), shown in the top plot. The black curve plots the average FAs that provide maximized signal amplitudes and distances for a given T_inv_. The corresponding signal amplitude and distance given T_inv_ and the optimized FA are shown (2nd and 3rd plots).

Incomplete inversion recovery balanced steady-state free precession (IIR-bSSFP) data acquisition was implemented in a 3D gradient-echo sequence that was modified by repeating nonselective inversion pulses followed by a fixed number (N) of segmented bSSFP acquisitions, where T_inv_ = N·TR is the time interval between an IR cycle (defined by two inversion pulses), and TR denotes the time of repetition (the schematic diagram shown in Figure 1a,b). A series of 3D images reconstructed at a series of inversion times (TIs) provided multi-contrast imaging. The simulated signal evolution curves of five representative brain tissues, including white matter (WM), grey matter (GM), cerebrospinal fluid (CSF), glioma, and edema are displayed in Figure 1c, given T1 and T2 values based on their reported values in previous studies at 3T [28,29,30]. The instantaneous signal evolution with IIR-bSSFP was derived based on Bloch equations [31] with a single-compartment model and tissue properties, by providing a combination of T1, T2, and MT contrast weightings.

### 2.2. Optimization of Scan Parameters

The scan parameters were optimized to achieve a good image quality and maximum contrast between adjacent tissue types by varying the inversion interval (T_inv_) and flip angle (FA) while holding constant routine imaging settings such as field of view, spatial resolution, and matrix size. Figure 1d shows the average signal amplitudes of all the tissue types, plotted as a 3D surface in purple, for a given a range of flip angles (10–60°) and T_inv_ (1–5 s). The average Euclidean distance between the signal evolution curves of the different tissues (blue surface) is also illustrated in Figure 1d, while Figure 1e demonstrates the tradeoff between maximizing signal amplitude (corresponding to the signal-to-noise level) versus distance (corresponding to tissue contrast). The average flip angles (black curve in Figure 1e) obtained based on maximizing signal amplitude (red curve) and signal difference (blue curve), respectively, resulted in an optimal choice of around FA = 30° for a range of T_inv_ between 2.5 and 4 s. Based on these joint simulation results, a T_inv_ of approximately 3 s was selected for this study, which was determined by the number of bSSFP readouts N times the actual TR, where maximal N readouts which can fit in T_inv_ are chosen.

### 2.3. Acceleration and Image Reconstruction

To achieve multi-contrast images from IIR-bSSFP acquisition, 3D k-space lines were segmented and distributed to the IR cycles. To improve data acquisition efficiency, a previously developed fast imaging technique, CIRcular Cartesian UnderSampling (CIRCUS) [32] was applied, which has been demonstrated in multiple applications [33,34,35,36,37]. CIRCUS integrates the desirable features of randomization, variable density, and flexible interleaving trajectories on a 3D Cartesian grid, to segment bSSFP acquisitions during the IR cycles. The CIRCUS sampling strategy was combined with k-t SPARSE-SENSE reconstruction [38,39] using a multi-coil compressed sensing reconstruction that exploited joint sparsity along the temporal dimension with a total variation constraint to achieve highly accelerated “dynamic” (multi-contrast) 3D imaging. A total of 20 frames (TIs) were chosen in the reconstruction (given T_inv_ of ~3 s), resulting in a reasonable and sufficient length of temporal footprint of 150 ms for each TI. Image reconstruction was implemented in MATLAB (The MathWorks, Natick, MA, USA) on high-performance servers (Four AMD Opteron 6380, 2.5 GHz, 256 GB Memory).

### 2.4. Brain Tissue and Lesion Segmentation

The most important preprocessing steps for conventional brain MRI segmentation include MRI bias field correction, image registration, and the removal of nonbrain tissue (so-called brain extraction or skull stripping). Although the images acquired with IIR-bSSFP also suffered non-uniformity in signal intensity, the shape and timing of the zero crossing during the inversion recovery of the signal evolution curves were independent of the absolute signal intensity. This allowed for the normalization of the signal evolution curve at each voxel by dividing by its norm, which removed spatial variations which were not due to underlying tissue properties. Multi-contrast imaging based on IIR-bSSFP acquisition provided efficient and reliable brain extraction and tissue segmentation. As this multi-contrast imaging was acquired as a single-scan, no registration was required among the multiple-contrast images. The resulting differential evolution curves for specific tissues also provided an alternative strategy (compared to traditional lesion segmentation methods) that did not rely on contrast between neighboring voxels at the tissue type boundary, which is often ill-defined.

**Figure 2:**
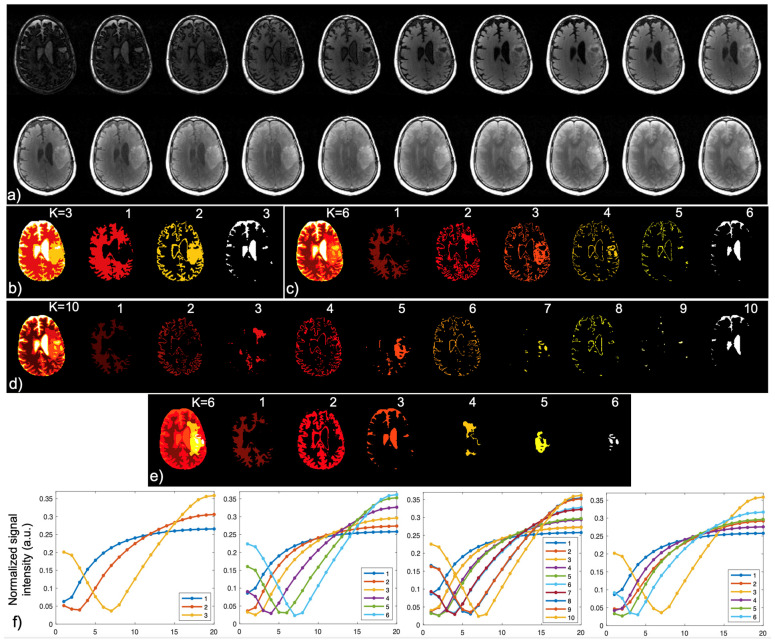
Automatic brain tissue segmentation and lesion detection based on the modified k-means clustering method achieved in this study. (**a**) Images acquired throughout inversion recovery (20 TIs); after signal intensity normalization and automatic skull removal, the following individual steps are applied with the modified k-means clustering approach: (**b**) three segments achieved, mainly presenting brain WM, GM, and CSF; (**c**) six segments achieved by further clustering each of the three segments in (**b**) into two segments; (**d**) total of ten segments achieved by further clustering each of the four segments #2–5 in (**c**) into two segments; (**e**) final segmentation of the brain tissues and lesions; and (**f**) the signal evolution curves of individual segments plotted for (**b**–**e**), respectively. WM: white matter; GM: grey matter; and CSF: cerebrospinal fluid.

A modified k-means clustering algorithm was developed for segmenting brain tissue based on the unique IIR-bSSFP acquisition by grouping the normalized signal evolution curve of each voxel to a cluster with the maximum inner product with respect to its center. Given the features of inversion recovery, the clusters were sorted by the time they reached a minimum signal of the signal evolution curve (reflecting the zero crossing during IR, apparently determined by the T1 values). From this modified k-means clustering method, an automatic brain extraction, tissue segmentation, and lesion detection workflow was developed, as shown in Figure 2.

Figure 2a displays the IIR-bSSFP images throughout the inversion recovery (20 TIs). Signal intensity normalization, as described above, compensates for signal variations across the image. With a signal intensity threshold of >2% to remove the background noise, three segments can be achieved using k-means clustering, mainly representing WM (along with skull), GM, and CSF. If a lesion exists, it will be clustered to the WM or GM during this step. The combination of the GM and CSF segments provides a rough brain contour for achieving automatic brain extraction (skull removal).

After skull removal, a sequential modified k-means clustering strategy was performed to both leverage the unique signal evolutions of IIR-bSSFP and reduce the partial volume effects that occur at tissue and lesion boundaries. This included the following: (1) clustering three segments (Figure 2b, mainly representing WM, GM, and CSF); (2) splitting each of them into two segments (Figure 2c, total of six segments, representing WM, four layers of GM, and CSF); and (3) further separating each of the four layers of GM into two segments (Figure 2d, representing mainly normal-appearing GM and potentially abnormal tissue, totaling ten segments). The final segmented brain regions (Figure 2e(#1–3), WM, GM, and CSF) and three lesions (Figure 2e(#4–6), two T2 lesions and the CEL) were automatically generated by merging the GM layers from Figure 2d(#2/4/6/8), combining the lesion segments in Figure 2d(#3/5/7), and deriving the CSF from Figure 2d(#9–10).

### 2.5. Two-Compartment Modeling and Multi-Parametric Mapping

**Figure 3:**
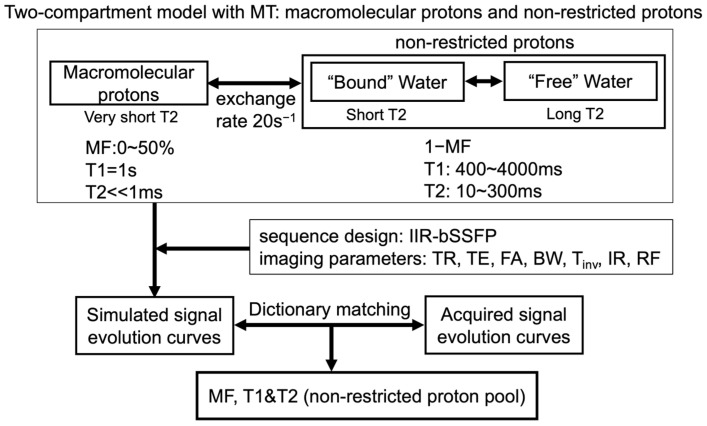
Two-compartment model built including macromolecular protons, non-restricted protons, and the exchange of magnetization between them. Signal evolutions are simulated based on the two-compartment model, given the sequence design (IIR-bSSFP), the imaging parameters, and assumptions on the tissue properties. The parameters MF, T1, and T2 are derived by matching the acquired signal evolution curves to the simulated ones.

Hydrogen proton distribution in the brain can be categorized into several compartments based on the protein-bound status of the protons, including the following: (1) those deep within the macromolecules with restricted motion (very short T2 << 1 ms, undetectable with conventional MRI methods); (2) water molecules bound to macromolecules (short T2 = 0~40 ms); (3) intracellular and extracellular water pools (intermediate T2 = 40~200 ms); and (4) free water pools (CSF, long T2 > 1 s) [40,41,42,43]. Macromolecular protons are usually undetectable with conventional MRI methods due to their short T2 (~10 us) but can be assessed via the MT effect, a well-known phenomenon during which an exchange of magnetization happens between bound water molecules with macromolecular protons and those in a free water pool [44,45,46]. As macromolecular proton fraction (MF) mapping derived from two-compartment modeling using MRI has been previously shown to correlate with myelin content in animal studies [47], a two-compartment model was constructed, which included macromolecular proton, non-restricted proton pools, and the exchange of magnetization between them (Figure 3). Signal evolutions were simulated based on the two-compartment model given the sequence design (IIR-bSSFP), the imaging parameters, and assumptions on tissue properties. Similar to the dictionary-searching method applied in MR Fingerprinting [48], the parameters MF, T1, and T2 were derived by matching the acquired signal evolution curves to the simulated ones (Figure 3).

### 2.6. Patient Scans

Fourteen patients (five females, 56.4 ± 10.4 years of age) with contrast-enhancing glioblastomas, scanned post surgery and chemoradiation with external beam radiation therapy and temozolomide, followed by additional targeted therapy if the tumor progressed, were recruited for this prospective, IRB-approved study. MRI examinations were performed on 3T MRI scanner (Discovery MR750; GE Medical Systems, Milwaukee, WI, USA) using a 32-channel phased-array head coil (Nova Medical, Wilmington, MA, USA). Whole-brain MRI using IIR-bSSFP acquisition with a 1mm isotropic resolution was acquired axially with a scan time of 6 min, a FOV (field of view) = 25.6 × 19.2 cm, an image matrix = 256 × 192 × 160, an FA = 30°, TR/TE (echo time) = 4.2/1.7 ms, BW (bandwidth) = 125 kHz, and T_inv_ = 3 s. The 3D images were reconstructed in a series of 20 TIs.

In addition to the multi-contrast IIR-bSSFP sequence, conventional 3D T2-FLAIR images with a 1 mm^3^ resolution using 3-fold parallel imaging acceleration (~8 min scan time) and pre- and post-contrast 3D T1-weighted IR-SPGR (spoiled gradient-recalled echo) images with a 1 mm^3^ resolution using 2-fold parallel imaging acceleration (~5 min each) were acquired and used to perform ground-truth lesion segmentation. The post-contrast 3D T1w IR-SPGR images were acquired ~3 min after the administration of 0.1 mmol/kg body weight of gadolinium contrast agent according to the standardized consensus protocol for brain tumor imaging [5].

In 6 of the 14 patients, conventional 2D T1 and T2 mapping were acquired on 6~8 slices centered at the lesion location, and 3D whole-brain myelin water fraction (MWF) mapping was acquired with MAGiC (SyntheticMR, Linköping, Sweden) [49]. The imaging settings for 2D T1 mapping were the following: spin-echo inversion recovery (SE-IR) sequence, TR/TE = 2550/10 ms, 4 TIs = 50/400/1100/2550 ms, an acceleration factor of R = 3, 6 slices of 3 mm thickness, and ~9 min scan time. Meanwhile, 2D T2 mapping was acquired with a Carl-Purcell–Meiboom-Gill (CPMG) sequence, TR = 1000 ms, 8 TEs = 25~200 ms (25 ms increment), R = 3, 8 slices of 3 mm thickness, and ~5 min scan time. MAGiC acquisition utilized a FOV = 25.6 × 20.5 cm, an isotropic resolution of 1.2 mm, an imaging matrix = 218 × 174 × 124, and a ~6 min scan time.

### 2.7. Data Analysis

In order to evaluate the accuracy of our segmentation method on IIR-bSSFP acquisition, the automated segmented regions of interest (ROIs) corresponding to T2L and CEL were compared to manually defined ROIs on conventional clinical images. Intra-exam image registration was first applied among the different scans using Slicer’s BrainsFit [50]. CEL and T2L ROIs were manually defined using clinically acquired structural MRI by A.J. (10 years of experience) using in-house software and further revised by either one of this study’s neuroradiologists, J.E.V.-M. (8 years of experience) or J.M.L. (22 years of experience), as necessary. Lesion volume was calculated and compared for each manually drawn and automatically segmented ROI. The parametric mapping values of each segmentation were calculated from IIR-bSSFP acquisition and compared to the reference methods (2D T1/T2 and MAGiC mapping).

## 3. Results

**Figure 4:**
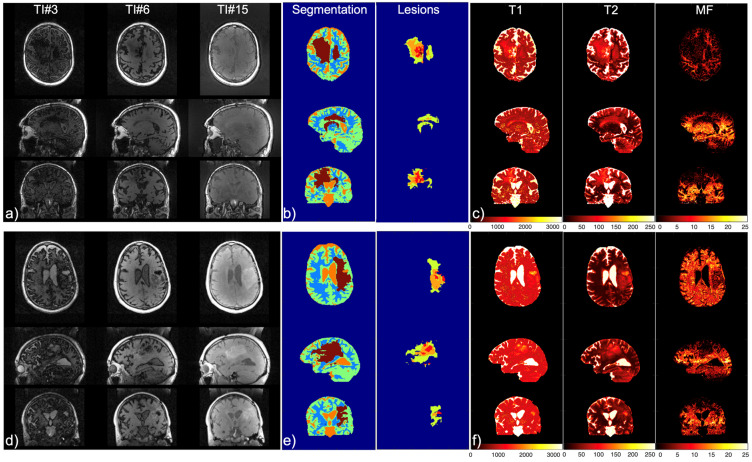
Images, segmentation, and maps of two patients. (**a**,**d**) Whole-brain multi-contrast imaging achieved with IIR-bSSFP acquisition, shown at three orthogonal reformats and three inversion times (3, 6, and 15 out of 20); (**b**,**e**) automatic segmentation and detection of brain tissues and lesions (right); and (**c**,**f**) T1, T2, and MF quantitative mapping derived from IIR-bSSFP using dictionary searching with the two-compartment model.

Figure 4 demonstrates the resulting three key parts of our method from two representative cases, including the whole-brain multi-contrast imaging achieved with IIR-bSSFP acquisition (representative images at three orthogonal reformats and three inversion times are shown in Figure 4a,d), the automatic segmentation of brain tissue and tumor lesions (shown in Figure 4b,e), and T1, T2, and MF quantitative maps (shown in Figure 4c,f). The pipeline was successfully applied on all 14 patients.

**Figure 5:**
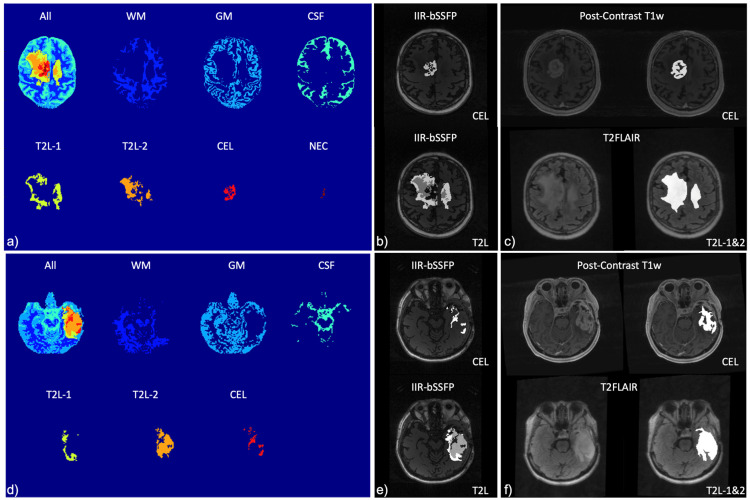
Automatic brain tissue and lesion segmentation from two patients. (**a**,**d**) Using the new method based on IIR-bSSFP acquisition; (**b**,**e**) the automatic lesion segments superimposed on brain anatomic images; and (**c**,**f**) manual segmentation of lesions using conventional imaging methods. WM: white matter; GM: grey matter; CSF: cerebrospinal fluid; CEL: contrast-enhancing lesion; T2L-1/-2: two regions of T2-hyperintense non-enhancing lesion; and NEC: necrosis.

Figure 5a,d show the results of the automatic tissue segmentation from two patient scans. Two T2L and one CEL segments were identified along with three typical brain tissue segments, WM, GM, and CSF, as well as necrosis (NEC) for the case shown in Figure 5a. Figure 5b,c,e,f display the results of the automatic brain lesion segmentation (overlaid on the structural images shown in Figure 5b,e) compared to the manual segmentation procedure from the conventional post-contrast T1w and T2 FLAIR images (such as the reference shown in Figure 5c,f).

**Figure 6:**
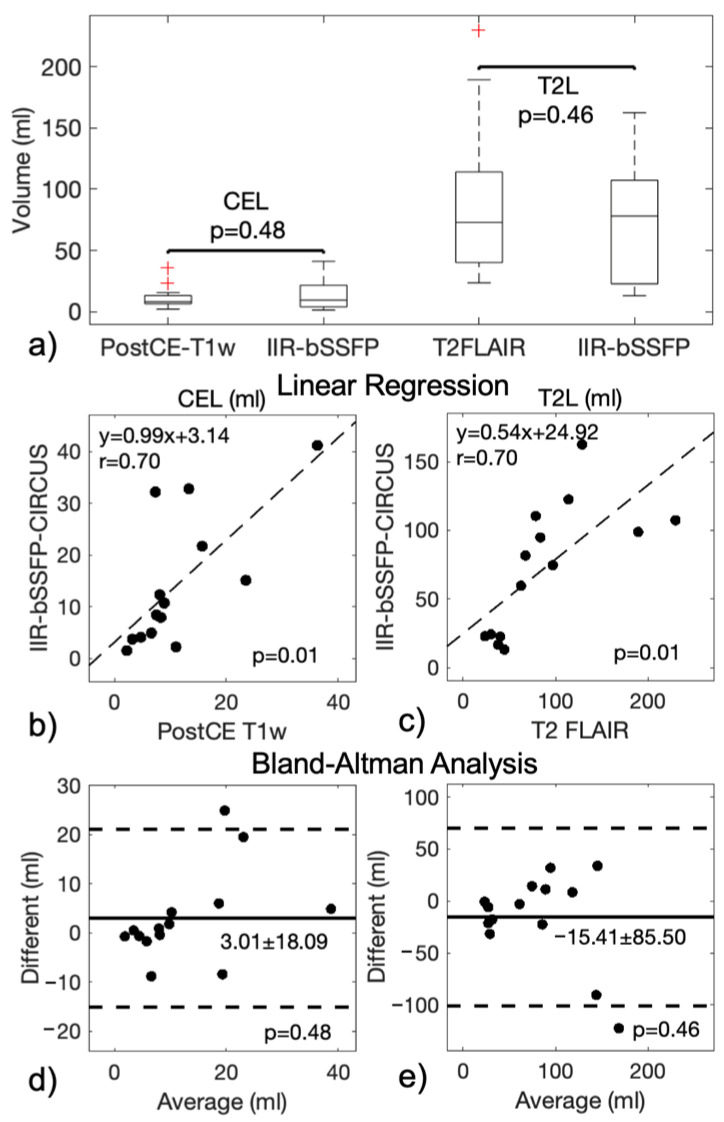
CEL and T2L volume measurements with the conventional methods and our developed method of IIR-bSSFP are plotted in (**a**), and the linear regression and Bland–Altman plots are shown in (**b**–**e**) (n = 14). Strong correlations (*p* < 0.05) of CEL and T2L volume measurements between the conventional and new methods were found; no significant difference (*p* > 0.05) in CEL and T2L volume measurements was found between the different methods (unit: mL).

As demonstrated in Figure 6, the volume measurements of CEL (14.1 ± 12.9 mL) and T2L (72.3 ± 46.9 mL) automatically obtained from the IIR-bSSFP sequence without a contrast injection were comparable to those manually defined on the reference post-contrast T1w (CEL 11.2 ± 9.1 mL) and T2 FLAIR (T2L 87.7 ± 60.9 mL) images, respectively (correlation coefficients r = 0.70, *p* < 0.01; paired *t*-test of differences in volumes *p* = 0.46 and 0.48 for CEL and T2L, respectively).

**Figure 7:**
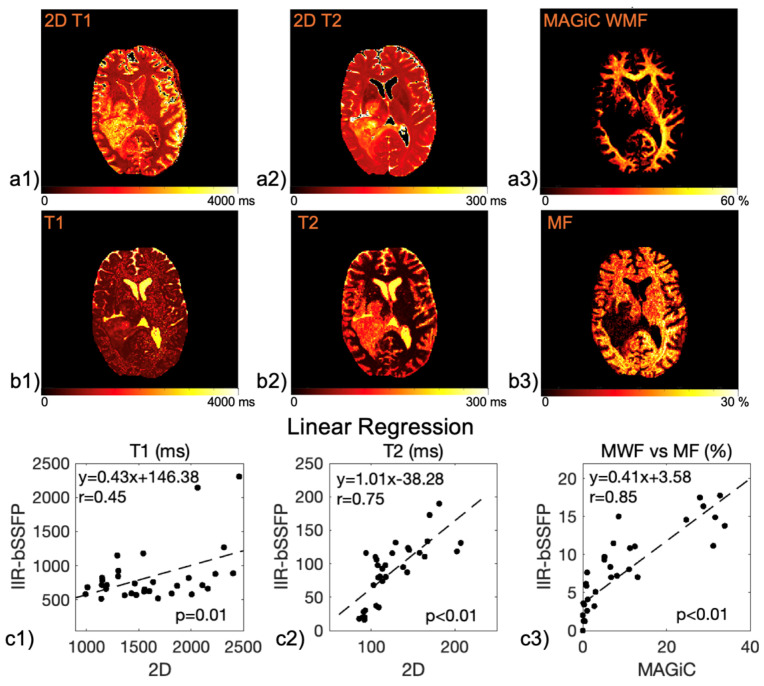
Parametric maps acquired on a representative patient with different imaging methods. (**a1**) 2D T1, (**a2**) 2D T2, (**a3**) 3D WMF with MAGiC, (**b1**–**b3**) 3D T1, T2, and MF maps with the new method, and (**c1**–**c3**) linear regression plots of the parametric values between different methods. Measurements of each tissue segment (excluding CSF) across four image slices averaged among six patient scans. T1 and T2 values found to be correlated between the new method and the reference 2D imaging (r = 0.45, *p* = 0.01; r = 0.75; *p* < 0.005), but with a significant difference between them (*t*-test, *p* < 0.05). A strong correlation (r = 0.85, *p* < 0.005) and no significant difference (*t*-test, *p* = 0.44) found between MF and MWF acquired with MAGiC.

Figure 7 shows the representative parametric maps acquired with 2D methods (Figure 7(a1–a2)), 3D MAGiC (Figure 7(a3)), and our method (Figure 7(b1–b3)). The parametric values in each tissue segment were averaged four image slices) and compared between the reference methods and the new method (six patient scans). The linear regression plots of the measurements (excluding CSF) are shown in Figure 7(c1–c3). The T1 and T2 measurements were found to be significantly correlated between our method and the reference 2D imaging (r = 0.45, *p* = 0.01; r = 0.75, *p* < 0.01, respectively), although they were statistically different (*t*-test, *p* < 0.01). MF was found to be both highly correlated with the MWF acquired with MAGiC (r = 0.85, *p* < 0.01) and not significantly statistically different (*t*-test, *p* = 0.44). Table 1 shows the measurements for each segment and method (four image slices from six patient cases were included for our analysis).

**Figure 8:**
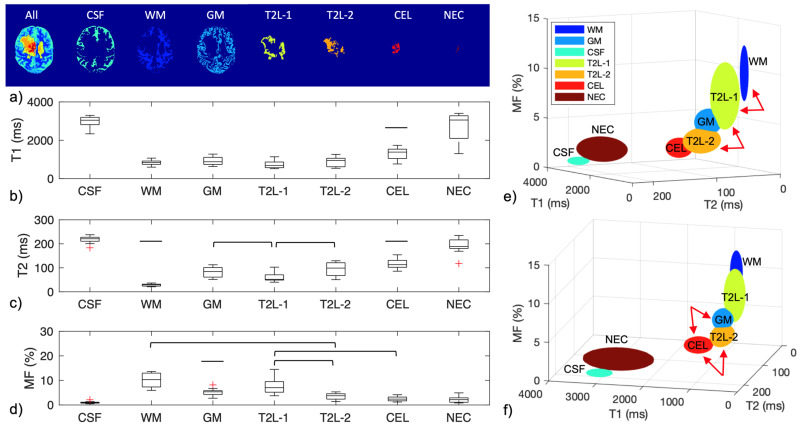
(**a**) Demonstration of brain tissue and lesion segmentation, (**b**) T1, (**c**) T2, and (**d**) MF measurements obtained with IIR-bSSFP. The differentiations with significant differences among the measurements are marked with bars (flat bars denoting significant differences between that measurement and all other ones). (**e**,**f**) 3D visualization of the T1-T2-MF mapping measurements of seven brain tissues and lesions at two orientations. The center and radius of the ellipsoid correspond to the mean and standard deviation of the measurements (whole-brain scan, n = 14).

Whole-brain multi-parametric mapping was achieved on all 14 patients using IIR-bSSFP. Figure 8b–d plot the three parametric values T1, T2, and MF of the whole brain for each segment (Figure 8a demonstrates the seven segments from a representative case) on all fourteen patient scans. Significant differences among the measurements are marked with bars on Figure 8b–d (flat bars denoting significant differences between that measurement and all other ones). As expected, the CEL had significantly increased T1 and T2 values compared to WM, GM, and T2 lesions (*t*-test, *p* < 0.05). Notice that two T2 lesions were detected with the new acquisition and automatic segmentation methods (Figure 2). The T2L-2, the more central portion of the T2L, had significantly decreased MF values compared to WM, GM, and T2L-1 (*t*-test, *p* < 0.05), similar to what had been observed in the CEL, likely suggesting the demarcation of an infiltrating tumor component. Table 2 shows the parametric mapping measurements in each segment (whole-brain images from 14 patient scans). Figure 8e,f show the 3D visualization with the T1/T2/MF measurements as the axes, where the center/radius of each ellipsoid corresponds to the mean/standard deviation of parametric map measurements. The arrows in Figure 8e,f demonstrate the ability to separate distinct lesion components from surrounding brain tissue without the use of a contrast agent, given their disguisable distributions on multi-parametric quantitative mapping.

## 4. Discussion

Despite parallel imaging capabilities and multichannel coils, the structural portion of clinical brain tumor imaging protocols typically takes up to 30 min and prohibits the acquisition of more biologically relevant physiological and metabolic imaging sequences. Furthermore, the provision of a single-scan strategy avoids the need for image registration and allows for reliable voxel-wise analyses within a lesion. Anatomic images with specific contrast can also be synthetized from the derived parametric maps, enabling an array of potential image contrasts to be retrospectively generated. Our unique strategy for automatically segmenting CEL and T2L regions without gadolinium-based contrast agents has the potential to improve clinical workflows by providing a rapid single-scan acquisition that allows more time for additional therapy-specific advanced imaging within a clinical MRI exam, avoiding potential gadolinium-based contrast deposition, and potentially more accurately delineating tumor cells within the T2 lesion. Although structural MRI has qualitatively relied on underlying tissue’s chemical composition, cellular tissue properties, and biophysical mechanisms, quantitative parametric mapping with MRI that includes the tissue’s chemical composition more accurately characterizes the underlying chemical and cellular properties of the imaged tissue, which, along with the automatically segmented shorter T2 component (T2L-2), potentially delineates infiltrating tumor margins beyond the CEL within T2-hyperintense edema. This potentially can help guide surgical resection, define radiation target volumes, and evaluate longitudinal changes in tumor burden and response to treatment.

This study uniquely employed IIR-bSSFP acquisition with CIRCUS undersampling for a high scan efficiency, along with the dictionary-matching strategy from MR Fingerprinting, to derive quantitative tissue parameters by matching the signal evolution acquired during IR to simulated instant evolution curves with the same scan parameters. Whereas MR Fingerprinting acquisitions involve deliberately varying MRI timing parameters such as flip angle and TR in a pseudorandom manner to generate separate signal evolutions (like fingerprints) for different tissues, our method employed a fixed flip angle and TR, combined with inversion recovery (T1 contrast) and bSSFP acquisition (T2-like contrast, involved with the MT effect), resulting in unique signal evolutions for different tissue types. While the Bloch equation can be used for simulating the signal evolution for a given flip angle, TR, TE, and the set of relaxation times for one single compartment, a model based on multiple compartments [29,51,52], including the magnetization exchange between the compartments within a voxel, more accurately simulates information about the tissue’s structure (such as MF). The model presented here was based on Bloch–McConnell equations [52,53] to generate signal evolution curves that included both macromolecular proton and free-water pools, as applied in Liu F et al. [52]. Although tissue characterization through the parametric mapping of relaxation parameters has a broad range of applications, conventional T1 and T2 maps are estimated separately by ignoring the tissue relaxation effect from the other component, resulting in inaccurate results. Simultaneous, multi-parametric mapping (such as T1/T2/proton density) in a single scan provides a more comprehensive approach to mapping, with an inherently improved accuracy, as has been achieved with bSSFP acquisitions during inversion recovery [54] and MR Fingerprinting methods which acquire non-steady-state signals after an inversion pulse [48]. The IR-bSSFP technique uses closed-form equations for calculating tissue parameters, while MR Fingerprinting derives tissue parameters by matching the acquired signal to a simulated signal evolution based on the Bloch equation. Since both methods require the full recovery of the magnetization after each inversion pulse and acquisition train, long scan times are a challenge, especially for 3D scans of tissues with long T1 relaxation times. Whereas MR Fingerprinting mitigates this drawback by highly accelerating data acquisition, fast T1 mapping can be achieved based on incomplete IR with continuous bSSFP acquisition, although it requires prior knowledge of the T2 values of the tissue, which limits its application [55].

Although the results presented demonstrate the high potential of this novel method for patients with gliomas, there are several limitations to this study. The first is the limited sample size given the preliminary nature of this feasibility study. The second is a lack of a real “gold standard” lesion volume for comparison, resulting in an apparent modest performance of our method (with correlation coefficient r = 0.70 to the reference method) due to inherent differences in the derived contrast-enhancing lesion volumes between our technique and the standard administration of gadolinium. As blood–brain barrier (BBB) breakdown results in the leakage of the contrast agent in the extravascular space of the surrounding tissue, resulting in T1 shortening and signal enhancement (CEL) on T1-weighted MR images, the leakage space is between the vasculature and the brain tissue where substances can enter, crossing the BBB breakdown. Although the detected CEL based on the enhancement reflects the leakage space, it could vary according to the contrast dose, the timing of the imaging with respect to the injection, and tissue permeability. As our method is not sensitive to these effects of the pooling of gadolinium into the extracellular–extravascular space over time, the CEL would be underestimated in these regions, especially for patients in which the time between injection and post-contrast scans is longer. Perfusion itself, however, also plays a critical role in explaining the observed variability, especially at the CEL margins [56], as high-grade gliomas are known for their heterogeneity in terms of both cerebral blood volume and permeability. As this technique is potentially more sensitive to the relaxation characteristics of the vasculature itself (including blood volume and permeability) than the clinical post-contrast T1-weighted scan, this would result in an overestimation of the lesion volumes in regions of elevated blood volumes or permeability. Future studies directly comparing our lesion segmentation with regions of high cerebral blood volumes and permeability are warranted to fully appreciate and understand these effects, as well as an investigation into which segmentations better correspond to the outcome, especially when monitoring response to therapy. In the presence of tumor cells (or treatment), brain tissue composition changes in terms of cellular heterogeneity, angiogenesis, necrosis, etc., which all alter the distribution of protons in the tissue and can change its T1, T2, and MF accordingly. Chemoradiation could complicate matters even further and potentially also influence BBB leakage without affecting T1, T2, and MF if the tissue microstructure is yet to be affected. As a result, there might not always be a close relationship between relaxation maps and BBB breakdown, potentially also contributing to some of the observed variability.

Although the T1 and T2 values measured with IIR-bSSFP and the two-compartment model varied compared to those obtained from conventional 2D T1 and T2 mapping measurements (Table 1 and Table 2; Figure 7), there is, in general, a lack of a ground-truth set of values, with variable T1 and T2 values reported throughout the literature depending upon the acquisition parameters, the coil, and the field strength employed, especially for those performing simultaneous multi-parametric mapping [57,58,59]. As a result, the 3D T1 and T2 maps generated with our two-compartment model had relatively low correlation coefficients (r = 0.45 and r = 0.75, respectively) when compared with those obtained with 2D methods based on single-compartment exponential fitting, despite reaching statistical significance (*p* < 0.05). Designed as a proof-of-principle study, a larger cohort is still needed for further validation of this novel approach, as well as studies assessing the benefits of simultaneous T1/T2/MF mapping compared to other physiologic and metabolic imaging metrics. Although bSSFP acquisitions can often suffer from banding artifacts, these were mitigated in this study by employing a relatively low flip angle (30°), short TR (~4 ms), and local shimming. Myelin water fraction mapping acquired with MAGiC was used a reference for evaluating our MF mapping because it was feasible to include within the time constraints of patient scans and still provided a relevant benchmark, even though it itself is not a gold standard. Finally, as the goal of this study was to focus on lesion delineation, future investigations are still warranted to determine the individual compartments of non-restricted protons (as shown in Figure 3) and the validity of the four GM layers that resulted from our clustering strategy (Figure 2) in terms of distribution, thickness, and tissue parametric mapping.

## 5. Conclusions

In conclusion, this 6 min, 1 mm isotropic, whole-brain scan and processing strategy was able to generate multi-contrast structural images and automatically segment the contrast-enhancing and T2-hyperintensity lesions of contrast-enhancing gliomas without the injection of a gadolinium-based contrast agent. In this preliminary study, the resulting lesion volume measurements were similar to those obtained with manual segmentation during conventional clinical structural imaging with contrast administration. Quantitative multi-parametric mapping further demonstrated the distinguishable lesion compartments within the T2-lesion. This approach has the potential to improve clinical workflow by (1) generating a measurement of the contrast-enhancing lesion burden in patients for whom gadolinium is not recommended (i.e., renal dysfunction), (2) substantially shortening the overall exam’s length, allowing more time for therapy-specific advanced imaging, and (3) providing a noninvasive, automatic tool for the longitudinal monitoring of response to therapy.

## Figures and Tables

**Table 1:** Parametric mapping measurements for each segment and imaging method (mean ± standard deviation; data from six patients, each with four acquired image slices; same data plotted in Figure 7). WM: white matter; GM: grey matter; T2L-1/-2: two regions of T2-hyperintense non-enhancing lesions; CEL: contrast-enhancing lesion; and NEC: necrosis.

Mapping	WM	GM	T2L-1	T2L-2	CEL	NEC
2D T1 (ms)	1186.4 ± 110.5	1589.5 ± 331.7	1451.5 ± 317	1657.9 ± 409.3	1947.8 ± 465.6	2022.3 ± 459.3
2D T2 (ms)	90.4 ± 2.7	117.5 ± 12.8	114.7 ± 14.2	138.2 ± 21.3	153.4 ± 47.6	152.0 ± 41.1
3D MWF (%)	30.4 ± 3.4	9.5 ± 3.3	12.5 ± 9.3	2.3 ± 1.8	0.55 ± 0.4	0.15 ± 0.0
IIR-bSSFP T1 (ms)	797.5 ± 88.3	670.4 ± 60.2	610.9 ± 58.1	598.4 ± 77.8	958.8 ± 204.9	1876.7 ± 608.8
IIR-bSSFP T2 (ms)	22.0 ± 5.7	85.2 ± 8.8	62.9 ± 26.5	115.4 ± 11.2	121.5 ± 10.1	157.5 ± 42.0
IIR-bSSFP MF (%)	14.9 ± 2.5	8.7 ± 1.9	11.9 ± 3.8	5.5 ± 2.6	3.6 ± 2.0	1.6 ± 0.5

**Table 2:** The parametric mapping measurements for each segment using IIR-bSSFP (mean ± standard deviation; data from 14 patient cases with whole-brain coverage; same data plotted in Figure 8). CSF: cerebrospinal fluid; WM: white matter; GM: grey matter; T2L-1/-2: two regions of T2-hyperintense non-enhancing lesion; CEL: contrast-enhancing lesion; and NEC: necrosis.

Mapping	CSF	WM	GM	T2L-1	T2L-2	CEL	NEC
IIR-bSSFP T1 (ms)	2990.4 ± 277.4	848.0 ± 135.5	912.4 ± 218.4	749.5 ± 217.2	883.8 ± 238.8	1300.7 ± 303.4	2696.6 ± 749.7
IIR-bSSFP T2 (ms)	218.1 ± 14.9	28.5 ± 5.3	83.1 ± 20.9	62.2 ± 22.4	94.1 ± 29.1	115.8 ± 19.1	190.7 ± 31.9
IIR-bSSFP MF (%)	1.0 ± 0.4	10.0 ± 2.8	5.3 ± 1.3	7.8 ± 3.4	3.4 ± 1.3	2.6 ± 1.0	2.2 ± 1.2

## Data Availability

Data were obtained at UCSF and are available upon request from and under the regulations of UCSF.
